# Inherited Metabolic Disorders Presenting with Ataxia

**DOI:** 10.3390/ijms21155519

**Published:** 2020-08-01

**Authors:** Grace Silver, Saadet Mercimek-Andrews

**Affiliations:** 1 Department of Pharmacology and Toxicology, University of Toronto, Toronto, ON M5S 1A8, Canada; grace.silver@mail.utoronto.ca; 2 Division of Clinical and Metabolic Genetics, Department of Paediatrics, The Hospital for Sick Children, Toronto, ON M5G 1X8, Canada; 3 Department of Paediatrics, University of Toronto, Toronto, ON M5G 1X8, Canada; 4 Genetics and Genome Biology Program, Research Institute, The Hospital for Sick Children, Toronto, ON M5G 1X8, Canada

**Keywords:** inherited metabolic disorders, ataxia

## Abstract

Ataxia is a common clinical feature in inherited metabolic disorders. There are more than 150 inherited metabolic disorders in patients presenting with ataxia in addition to global developmental delay, encephalopathy episodes, a history of developmental regression, coarse facial features, seizures, and other types of movement disorders. Seizures and a history of developmental regression especially are important clinical denominators to consider an underlying inherited metabolic disorder in a patient with ataxia. Some of the inherited metabolic disorders have disease specific treatments to improve outcomes or prevent early death. Early diagnosis and treatment affect positive neurodevelopmental outcomes, so it is important to think of inherited metabolic disorders in the differential diagnosis of ataxia.

## 1. Introduction

Coordination and balance are controlled by a complex network system including the basal ganglia, cerebellum, cerebral cortex, peripheral motor, and sensory pathways. The cerebellum contributes to coordination, quality of movement, and cognition. There are a large number of inherited metabolic disorders affecting the cerebellum and resulting in cerebellar atrophy or hypoplasia [1,2].

Ataxia is described as abnormal coordination secondary to cerebellar dysfunction, vestibular dysfunction, or sensorial dysfunction. Ataxia can present as gait ataxia, truncal ataxia, tremor, or nystagmus depending on the involved parts of the nervous system [3,4]. All types of ataxia can present individually or in combination in a single patient. Ataxia can be an important part of the clinical picture in inherited metabolic disorders which can guide physicians to targeted investigations to identify underlying causes. This is particularly important for diagnosing treatable inherited metabolic disorders to improve neurodevelopmental outcomes.

Inherited metabolic disorders are individually rare, but their collective prevalence is about 1 in 1000 live births. Inborn Errors of Metabolism Knowledgebase listed more than 150 inherited metabolic disorders presenting with ataxia (http://www.iembase.org/gamuts/store/docs/Movement_disorders_in_inherited_metabolic_disorders.pdf, accessed on 29 May 2020) [5].

**Figure 1:**
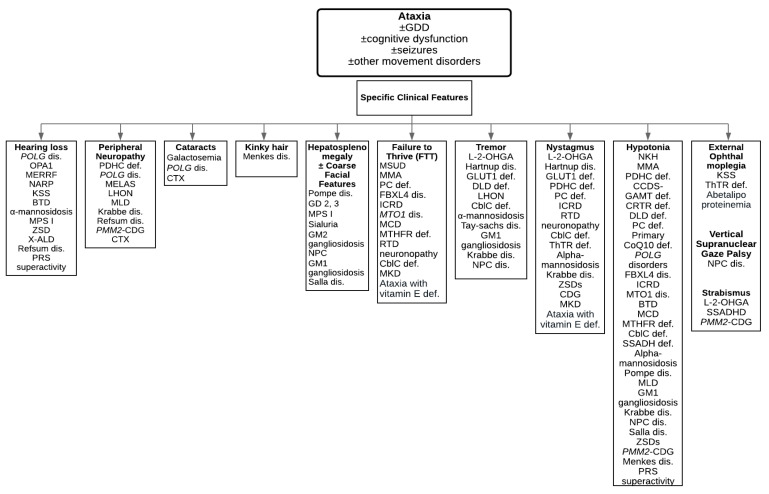
A diagnostic algorithm to guide physicians towards characteristic clinical features. Abbreviations: BTD = Biotinidase deficiency; cblC def = Cobalamin C deficiency; CCDS = Cerebral creatine deficiency syndromes; DLD def = Dihydrolipoamide dehydrogenase deficiency; GLUT1 def = Glucose transporter 1 deficiency; ICRD = Infantile cerebellar-retinal degeneration; KSS = Kearns-Sayre syndrome; L-2-OHGA = L-2-hydroxyglutaric aciduria; LHON = Leber hereditary optic neuropathy; MCD = Multiple carboxylase deficiency; MKD = Mevalonate kinase deficiency; MLD = Metachromatic leukodystrophy; MMA = Methylmalonic acidemia; MSUD = Maple syrup urine disease; MTHFR def = Methylenetetrahydrofolate reductase deficiency; NKH = Nonketotic hyperglycinemia; NPC dis = Niemann-Pick type C disease; PC def = Pyruvate carboxylase deficiency; PDHC def = Pyruvate dehydrogenase complex deficiency; PMM2-CDG = Phosphomannomutase 2-Congenital disorder of glycosylation; PRS superactivity = Phosphoribosylpyrophosphate synthetase superactivity; Primary CoQ10 def = Primary coenzyme Q10 deficiency; RTD = Riboflavin transporter deficiency; SSADH def = Succinic semialdehyde dehydrogenase deficiency; ThTR def = Thiamine transporter deficiency; ZSDs = Zellweger spectrum disorders.

Ataxia is usually part of a complex phenotype in inherited metabolic disorders. There are several important features and signs to point towards inherited metabolic disorders (Figure 1). In medical history, the presence of recurrent somnolence and coma episodes during intercurrent illnesses, a history of protein aversion or failure to thrive, a history of progressive loss of skills, hearing loss, hair abnormalities, behavioral problems, and seizures in combination with ataxia and global developmental delay can guide physicians to consider inherited metabolic disorders in their differential diagnosis. In family history, global developmental delay, cognitive dysfunction, psychiatric disorders, recurrent miscarriage, sudden infant death syndrome, and congenital malformations in other family members can be important clues to consider inherited metabolic disorders in the differential diagnosis. Some of the clinical features together with ataxia can suggest inherited metabolic disorders such as hepatosplenomegaly (lysosomal storage disorders), cardiomyopathy (lysosomal storage disorders, mitochondrial disorders, congenital disorders of glycosylation (CDG)), complex movement disorders (mitochondrial disorders, organic acidurias, neurotransmitter disorders), macrocephaly (Canavan disease, lysosomal storage disorders), or microcephaly (glucose transporter 1 (GLUT1) deficiency, neuronal ceroid lipofuscinosis ((NCL)). To investigate the underlying causes, detailed medical history, family history, and physical examination are important to obtain.

Some of the inherited metabolic disorders can present with episodic or intermittent ataxia during intercurrent illness, stress situations, or prolonged fasting. This is due to an increased energy demand and decreased energy production due to the defects in energy metabolism pathways and in the production or transport of fuels such as glucose and ketone bodies, or the increased production of toxic metabolites such as amino acids or organic acids secondary to catabolism. Episodic or intermittent ataxia can be observed in maple syrup urine disease, pyruvate dehydrogenase complex (PDHC) deficiency, and GLUT1 deficiency. In progressive neurodegenerative disorders, e.g., lysosomal storage disorders, ataxia is usually progressive. In energy metabolism disorders, ataxia is usually static with acute episodes of deterioration during intercurrent illnesses. It is important to remember these disorders to initiate diagnostic investigations and start appropriate treatment.

Recently, we reported the genetic landscape of pediatric movement disorders. Ataxia was the most common movement disorder in 53% of the patients who underwent genetic investigation in our clinic [6]. Interestingly, 74% of patients with ataxia had 16 different underlying genetic diseases. There were 8 different inherited metabolic disorders including GLUT1 deficiency, *SURF1* Leigh’s disease, PDHC deficiency, *DNAJC19* disease, *ND3* mitochondrial encephalopathy, NCL type 2, riboflavin transporter deficiency, and 3-hydroxy-3-methylglutaryl CoA synthase 2 deficiency [6]. Seizure and a history of developmental regression are especially important clinical denominators when considering the underlying inherited metabolic disorders in patients with ataxia.

Cerebellar atrophy in brain magnetic resonance imaging (MRI) can be found in mitochondrial disorders (e.g., mitochondrial encephalomyopathy, lactic acidosis, and stroke-like episodes (MELAS), neuropathy, ataxia, and retinitis pigmentosa (NARP), Kearns–Sayre syndrome, leukoencephalopathy with brain stem, spinal cord involvement, and lactate elevation, PDHC deficiency, primary coenzyme Q10 deficiency, *POLG* disease), lysosomal storage disorders (metachromatic leukodystrophy (MLD), GM2 gangliosidosis, Niemann–Pick type C (NPC), Salla disease, NCL, fatty acid hydroxylase-associated neurodegeneration), classical galactosemia, L-2-hydroxyglutaric aciduria, Menkes disease, and mevalonate kinase deficiency [1,2].

Table 1 summarizes the vast majority of inherited metabolic disorders presenting with ataxia, including category of inherited metabolic disorders, genes, and clinical features. Table 2 lists metabolic investigations. Disease-specific treatments to improve outcomes in inherited metabolic disorders are summarized in Table 3 [5,7,8,9,10,11,12,13,14,15,16,17,18,19,20,21,22,23,24,25,26,27,28,29,30,31,32,33,34,35]. We provide a list of disorders based on the specific clinical features in Figure 1 [36]. In this review article, we list some of the treatable inherited metabolic disorders in details below.

## 2. Treatable Inherited Metabolic Disorders Presenting with Ataxia

### 2.1. Disorders of Amino Acid Metabolism and Transport

#### Maple Syrup Urine Disease (MSUD)

Maple syrup urine disease (MSUD) is an autosomal recessive disorder of branched chain amino acid (leucine, valine, isoleucine) catabolism due to branched-chain alpha-ketoacid dehydrogenase complex (BCKD) deficiency encoded by *BCKDHA*, *BCKDHB*, and *DBT* genes [7,8]. The BCKD complex deficiency results in the accumulation of leucine, valine, isoleucine, and alloisoleucine [7,9]. Its estimated incidence is 1 in 185,000 live births, with a higher prevalence of up to 1 in 113 in Ashkenazi Jewish populations due to a founder pathogenic variant in *BCKDHB* (c.548G > C) [7].

Phenotypes of MSUD consist of classical, intermediate, intermittent, and thiamine responsive [7,8]. The classical MSUD, or severe neonatal onset form, presents in the first week of life with feeding intolerance, encephalopathy, and seizures. Characteristic maple syrup odor can be present in cerumen as early as 12 hours [7,8,9]. If left untreated, the severe neonatal onset form may progress to coma or death secondary to brain edema [8]. Intermediate and intermittent forms typically manifest during any age ranging from infantile to adulthood. Clinical features of the intermediate form include global developmental delay, failure to thrive, maple syrup urine smell, and intermittent episodes of encephalopathy during intercurrent illnesses [7,8]. Usually, patients with the intermittent form may have normal early development and growth and can be missed until a catabolic state or intercurrent febrile illness when ataxia may manifest as an initial presentation [7,8]. The clinical presentation of the thiamine responsive form is similar to the intermediate form and is responsive to thiamine supplementation.

In the neonatal period, the acute elevation of leucine levels results in fencing and bicycling movements, irritability, and opisthotonos. In older children, the acute elevation of leucine levels results in acute neurological deterioration characterized by an altered level of consciousness, acute dystonia, and ataxia which may progress to coma. Hyperactivity, sleep disturbances, and hallucinations are also reported.

Patients with classical MSUD can be identified by positive newborn screening for MSUD or with the above symptoms. Plasma amino acid analysis reveals elevated leucine, alloisoleucine, valine, and isoleucine. Elevated ketones may cause metabolic acidosis. Ammonia is usually normal in MSUD patients but may be mild to moderately elevated during acute metabolic decompensations in some patients [9]. Intermediate and intermittent forms are often missed by newborn screening due to higher BCKD activity resulting in initial normal leucine plus isoleucine levels [7,8]. Plasma amino acid analysis can be normal or mildly elevated in intermittent forms of MSUD outside of metabolic decompensations. In patients with episodes of ataxia during intercurrent illness, plasma amino acid analysis is highly recommended; if plasma amino acid analysis is not collected during an acute metabolic decompensation, the intermittent form of MSUD can be missed.

Treatment consists of a dietary restriction of leucine, supplementation of valine and isoleucine, and branched chain amino acid free medical formula. During intercurrent illness, caloric intake should be increased to prevent catabolism and leucine elevation, which could result in acute encephalopathy and brain edema [7,8]. If medical treatment is not successful in decreasing leucine levels, hemodialysis is undertaken to remove leucine and prevent coma and death due to leucine toxicity [9]. If patients require strict leucine restriction and several hospital admissions during intercurrent illnesses, non-related orthotopic liver transplantation is the treatment of choice [9].

### 2.2. Disorders of Carbohydrate Metabolism

#### 2.2.1. Galactose-1-phosphate Uridylyltransferase Deficiency

Galactose-1-phosphate uridylyltransferase (GALT) deficiency is an autosomal recessive disorder of the galactose metabolism. Galactosemia is due to biallelic pathogenic variants in *GALT* [10]. Its prevalence is 1 in 48,000 as per the National Newborn Screening and Genetics Resources Center. It is more common in Ireland with a prevalence of 1 in 16,476 [11,12]. GALT deficiency results in the accumulation of galactose, galactose-1-phosphate, and galactitol [10,11,12].

Classical galactosemia patients present in the neonatal period with feeding problems, hepatic failure, and coagulopathy that can acutely progress to multi organ failure and potentially death if untreated [10,11,12]. Despite adequate treatment with galactose restricted diet, patients can suffer from developmental delay, speech delay, and motor dysfunction presenting with ataxia and tremor. Females are at risk of gonadal dysfunction and premature ovarian insufficiency [11,12].

The long-term neurodevelopmental outcome is characterized by speech problems, learning difficulties, and cognitive dysfunction even in treated patients. A small number of patients present with tremor, either intentional or postural, cerebellar ataxia, and dystonia.

The National Newborn Screening Programs included galactosemia in their list of disorders to identify and treat newborns early. Diagnosis is confirmed by the measurement of erythrocyte galactose-1-phosphate concentration or reduced GALT enzyme activity [11]. Treatment consists of dietary lactose and galactose restriction [10].

#### 2.2.2. Glucose Transporter 1 (GLUT1) Deficiency

Glucose is transported from the bloodstream to the central nervous system by glucose transporter 1 (GLUT1), encoded by *SLC2A1.* A genetic defect in this transporter protein results in impaired glucose supply to the brain, affecting brain development and function, called GLUT1 deficiency. Since its first description, there have been about 400 patients reported in the literature [13]. It is an autosomal dominant disorder caused by heterozygous pathogenic or likely pathogenic variants in *SLC2A1*.

The phenotype ranges from early onset severe global developmental delay, epileptic encephalopathy, acquired microcephaly, ataxia, dystonia, and spasticity to paroxysmal movement disorder including intermittent ataxia, choreoathetosis, dystonia, and alternating hemiplegia with or without cognitive dysfunction or intellectual disability. In a study, 57 patients with GLUT1 deficiency were reported with their distribution of movement disorders. Ataxia and ataxia plus spastic gait were the most common movement disorder in 70% of the patients. Ataxia improved after feeding in some of the patients [14].

The characteristic biomarker is low cerebrospinal fluid (CSF) glucose or low CSF to blood glucose ratio in the presence of normal blood glucose. Blood glucose should be collected within 30 min prior to lumbar puncture. If blood glucose is not collected, the CSF glucose level is also helpful to guide the diagnosis of GLUT1 deficiency. The CSF-to-blood glucose ratio ranges between 0.19 and 059 and the CSF glucose ranges between 0.9 and 2.88 mmol/L in patients with GLUT1 deficiency [13]. The diagnosis is confirmed by either a genetic test using targeted next generation sequencing for epilepsy, movement disorders, intellectual disability, or whole exome sequencing. In patients with low CSF glucose levels, direct Sanger sequencing of *SLC2A1* can be applied to confirm the diagnosis [13]. In about 90% of patients, diagnosis is confirmed by sequence analysis and in about 10% of the patients, a deletion/duplication test is important to apply to identify small deletion and duplications.

The ketogenic diet has been the gold standard for the treatment of GLUT1 deficiency. The response to treatment varies depending on the age of diagnosis and application of the ketogenic diet. About 65% of patients become seizure free in one week to one month of the ketogenic diet therapy. It is recommended that beta-hydroxybutyrate levels are kept at 4–5 mM. The response to the ketogenic diet is variable.

### 2.3. Disorder of Mitochondrial Energy Metabolism

#### 2.3.1. Creatine Deficiency Disorders

Arginine, glycine amino acid, L-arginine:glycine amidinotransferase (AGAT), and guanidinoacetate N-methyltransferase (GAMT) are involved in creatine synthesis in the kidney and liver. After synthesis, creatine is taken up by high energy demanding organs, such as the brain, muscles, and the retina by an active sodium chloride dependent creatine transporter (CRTR). There are three disorders involving synthesis and transport of creatine, including AGAT, GAMT, and CRTR deficiencies. The first two enzyme deficiencies are inherited autosomal recessively while CRTR deficiency is an X-linked disorder. They are ultra-rare disorders with less than 130 patients with GAMT deficiency, less than 20 patients with AGAT deficiency, and less than 200 patients with CRTR deficiency reported so far [15].

Global developmental delay and cognitive dysfunction are the most common clinical features and present in all untreated patients in the three creatine deficiency disorders. Epilepsy, movement disorders, and behavioral problems are observed in GAMT and CRTR deficiencies. The phenotype ranges from asymptomatic to severe phenotypes in females in CRTR deficiency. GAMT deficiency leads to complex movement disorders in combination with ataxia and tremor; choreoathetosis and dystonia; dystonia, chorea, and ataxia; myoclonus and bradykinesia; or ballismus and dystonia [16]. In a study, 50% of the patients who were older than 6 years of age at the time of the diagnosis of GAMT deficiency presented with ataxia [17]. In 101 male patients with CRTR deficiency, a wide based gait, ataxia, dysarthria, and coordination problems were reported in 29% of them [18].

The biochemical hallmarks are cerebral creatine deficiency in brain magnetic resonance spectroscopy (^1^H-MRS) in GAMT and AGAT deficiencies as well as in males with CRTR deficiency. Urine, plasma, and cerebrospinal fluid (CSF) guanidinoacetate is elevated in GAMT deficiency, low in AGAT deficiency, and normal in CRTR deficiency. The urine creatine is elevated in males with CRTR deficiency. Females can have a normal or mildly elevated urine creatine. In the presence of abnormal biochemical features, Sanger sequencing of *GAMT, GATM* or *SLC6A8* confirms the diagnosis of these disorders [15].

Creatine supplementation is applied in all creatine deficiency disorders. Ornithine supplementation and protein- or arginine-restricted diet are applied in GAMT deficiency. Arginine and glycine supplementation are applied in CRTR deficiency [15]. Epilepsy improves in about two-thirds of the patients with GAMT deficiency and movement disorder improves in about 50% of the patients with GAMT deficiency.

#### 2.3.2. Primary Coenzyme Q10 Deficiency

Coenzyme Q10 is an essential cofactor involved in various cellular pathways including the electron transport chain, the beta oxidation of fatty acids, and pyrimidine biosynthesis [19]. There are more than 10 genetic defects involved in the coenzyme Q10 biosynthesis causing primary coenzyme Q10 deficiency (Table 1) [19,20]. The genes associated with primary coenzyme Q10 deficiency are listed in Table 1. Inherited primary coenzyme Q10 deficiency disorders are autosomal recessive disorders [20].

The clinical features of primary coenzyme Q10 deficiency are complex and involve multiple organs or systems including global developmental delay, cognitive dysfunction, seizures, ataxia, movement disorder, spasticity, cardiomyopathy, hearing loss, peripheral neuropathy, and retinopathy [19,20].

A definitive biochemical diagnosis is confirmed by deficient coenzyme Q10 amounts in muscle biopsy specimen and skin fibroblasts [19,20]. The biochemical diagnosis is confirmed by either targeted next generation sequencing panel for coenzyme Q10 deficiency or by whole exome sequencing.

Treatment consists of oral coenzyme Q10 supplementation, which is well tolerated. The response to the treatment is variable and treatment outcome data is sparse [19,20].

### 2.4. Vitamin and Cofactor Responsive Disorders

#### 2.4.1. Biotinidase Deficiency

Biotinidase deficiency is an autosomal recessive disorder due to the biallelic pathogenic variants in *BTD*, encoding biotinidase [21]. Biotinidase is responsible for the recycling of biotin from biocytin to contribute to the free biotin pool. Biotin is an important cofactor for the conversion of apocarboxylases to holocarboxylases, including pyruvate carboxylase, 3-methylcrotonyl-CoA carboxylase, propionyl CoA carboxylase, and acetyl-CoA carboxylase [21,22]. Biotinidase deficiency is sometimes referred to as late onset multiple carboxylase deficiency, as biotin deficiency impacts numerous enzymatic processes [22]. Its estimated prevalence is 1 in 60,000 worldwide, including 1 in 137,000 for profound and 1 in 110,000 for partial biotinidase deficiency [22,23]. In some expanded newborn screening programs, biotinidase deficiency is included to identify newborns in the asymptomatic stage.

Profound biotinidase deficiency can present with seizures, alopecia, hypotonia, and skin rash in the first few months of life. If left untreated, patients present with global developmental delay, ataxia, optic atrophy, ophthalmological problems, and hearing loss [21,22,23]. Older patients with later onset tend to display ataxia and movement disorders as initial clinical presentations [23].

Untreated biotinidase deficiency has numerous biochemical abnormalities including metabolic acidosis, hyperammonemia, or lactic acidosis which may present as an acute metabolic decompensation during intercurrent illness [22]. An abnormal acylcarnitine profile and urine organic acid analysis are suggestive and biochemical diagnosis is confirmed by deficient biotinidase activity in serum. If the biotinidase activity is less than 10% normal, it is called profound deficiency. If the biotinidase activity is 10–30% normal, it is called partial deficiency [23].

Treatment consists of lifelong biotin supplementation, resolving seizures, ataxia, skin rash, and alopecia quickly [21,22]. If hearing loss, vision problems, or severe developmental delay present, biotin supplementation will not resolve them, indicating the importance of early intervention [21,22,23].

#### 2.4.2. Riboflavin Transporter Deficiency

Riboflavin is a precursor of flavin mononucleotide (FMN) and flavin adenine dinucleotide (FAD), which are crucial cofactors for the electron transport chain and beta oxidation of fatty acids in the mitochondria for energy production. To maintain riboflavin metabolism in the human body, riboflavin is transported across membranes using riboflavin transporters including RFVT1, encoded by *SLC52A1*, RFVT2, encoded by *SLC52A*2 and RFVT3, encoded by *SLC52A3.* Biallelic pathogenic variants in *SLC52A2* and *SLC52A3* are reported in human disease after 2010. Both genetic defects are associated with previously known Brown–Vialetto–Van Laere (BVVL) and Fazio–Londe (FL) syndromes [24].

Disease onset ranges from early infantile onset to early adulthood onset. Usually, a history of developmental regression is the initial symptom. Symptoms range from motor dysfunction, muscle weakness, hypotonia, ataxia, failure to thrive, or hearing loss to peripheral neuropathy. Bulbar symptoms, respiratory failure, and optic atrophy are also common features. These are progressive disorders, if untreated.

Diagnosis is suspected by acylcarnitine profile and urine organic acid abnormalities resembling multiple acyl-CoA dehydrogenase deficiency or ethylmalonic aciduria. Complex II deficiency in muscle biopsy has been reported in two patients with riboflavin transporter deficiency [25].

Diagnosis is confirmed either by the direct Sanger sequencing of *SLC52A2* and *SLC52A3* genes or the application of targeted next generation sequencing panels for nuclear mitochondrial disorders or whole exome sequencing.

High dose oral riboflavin supplementation is the treatment. The timing of treatment onset is essential for favorable treatment outcomes [24,25].

### 2.5. Organelle Related Disorders: Lysosomal Storage Disorders

#### Neuronal Ceroid Lipofuscinosis (NCL)

Neuronal ceroid lipofuscinoses (NCL) are the most common lysosomal storage disorders with an estimated prevalence of 1.5 to 9 per a million people [26]. There are more than 10 subtypes. The most common subtypes are type 2, *CLN2,* and type 3, *CLN3* diseases. Disease onset ranges from infantile to adulthood even within the same subtype.

Clinical features are characterized by a history of developmental regression in motor and cognitive functions, seizures, visual problems, and early death [27]. Progressive ataxia is a common feature in NCL secondary to progressive cerebellar atrophy [6]. The first symptom is usually a seizure which is followed by developmental regression. Motor dysfunction is characterized by myoclonus in infants and ataxia and spasticity in older children.

Three of the NCL genes encode an enzyme, cathepsin D, encoded by *CTSD*; palmitoyl-protein thioesterase, encoded by *PPT1* or *CLN1*, and tripeptidylpeptidase 1, encoded by *TPP1* or *CLN2* [28]. Granular osmiophilic, curvilinear, and fingerprint lipopigments identified by the electron microscopic examination of lymphocytes, skin cells, or cells from conjunctival biopsy are the suggestive neuropathological features of NCL. The diagnosis is confirmed by targeted Sanger sequencing, the application of an NCL panel, or whole exome sequencing. Many NCL genes are also included in targeted next generation sequencing panel for epilepsy [29].

Treatment is symptomatic for the majority of NCL. Recently, intracerebroventricular cerliponase alfa infusions for the treatment of *CLN2 (TPP1)* disease was approved for clinical use [27,30]. Trials for *CLN3* and *CLN6* are underway in clinical Phase I/II human studies.

### 2.6. Organelle Related Disorders: Peroxisomal Disorders

#### 2.6.1. X-linked adrenoleukodystrophy (X-ALD)

X-linked adrenoleukodystrophy (X-ALD) is due to hemizygous pathogenic variants in *ABCD1*, which encodes ATP-binding cassette domain 1 (ABCD1). This protein is a peroxisome transporter protein. Due to the pathogenic variants and ABCD1 dysfunction, very long chain fatty acids accumulate, especially in the adrenal glands and the brain. Its estimated prevalence is 1 in 20,000 males [31].

The age of symptom onset is variable and ranges from childhood to adulthood. The most severe form is the childhood onset cerebral form. After normal cognitive and motor function, males present with behavioral problems, followed by progressive motor and cognitive dysfunction and progressive ataxia between the ages of 4 and 8 years, leading to complete motor and cognitive dysfunction within a few years. The cerebral form can also be seen in adolescents and adults with a slower disease progression [31]. Adrenal insufficiency is a common feature requiring hormone replacement therapy. Adrenomyeloneuropathy is the most common phenotype, with spinal cord disease leading to chronic progressive spasticity, neuropathy, and incontinence [31,32].

The diagnosis is suspected by characteristic brain MRI features including symmetrical increased signal intensity in parieto-occipital region in T2 weighted images. Active demyelination areas are enhanced with contrast. The biochemical hallmark is elevated plasma very long chain fatty acids. The diagnosis is confirmed by direct sequencing and deletion/duplication analysis of *ABCD1* [31,32].

The treatment is hematopoietic stem cell transplantation (HSCT) in males, with disease progression stopping after HSCT. The decision for HSCT depends on disease morbidity. In males with extensive white matter changes, motor, and cognitive dysfunction, HSCT is not recommended [31,32].

#### 2.6.2. Refsum Disease

Refsum disease is called classic or adult Refsum disease. This is an autosomal recessively inherited peroxisomal disease due to biallelic variants in *PHYH* (>90%, encodes phytanoyl-CoA hydroxylase [35].

The age of onset ranges from 7 months to older than 50 years. Retinitis pigmentosa and anosmia are early clinical features. About 10–15 years later, neuropathy, ataxia, muscle weakness, sensory loss, deafness, and ichthyosis are the part of the clinical features. Initially, patients present with an unsteady gait which progresses to ataxia as a result of cerebellar dysfunction. Additionally, motor and sensory polyneuropathy, skeletal abnormalities, and cardiac arrhythmias have been reported [35].

Elevated plasma phytanic acid and pipecolic acid suggest the diagnosis of Refsum disease. The diagnosis is confirmed by direct Sanger sequencing of *PHYH* [35].

The treatment consists of phytanic acid restricted diet. This diet can improve polyneuropathy, ataxia and ichthyosis and arrhythmias due to decrease in plasma phytanic acid levels. In acute onset arrhythmias and severe weakness, plasmapheresis or lipopheresis can be used to improve symptoms. Weight loss or decreased caloric intake mobilizes stored lipids and causes the acute deterioration of symptoms, which should be avoided by a high caloric intake diet [35].

## 3. Conclusions

Inherited metabolic disorders are individually rare. Some inherited metabolic disorders have disease-specific treatments to improve neurodevelopmental outcomes and to prevent early death. It is important to think of inherited metabolic disorders in the differential diagnosis of ataxia.

A medical history of somnolence and coma during intercurrent illness, the progressive loss of skills, hearing loss, behavioral problems, seizures, and global developmental delay are important to suggest inherited metabolic disorder. A history of developmental regression is an important symptom to suggest lysosomal storage disorders. Detailed three-generation family history can help identifying X-linked, autosomal dominant, or mitochondrial disorders. A family history of global developmental delay, cognitive dysfunction, psychiatric disorders, recurrent miscarriage, sudden infant death syndrome, and congenital malformations can suggest inherited metabolic disorders. A detailed review of neuroimaging can identify specific neuroimaging features to suggest specific inherited metabolic disease. For example, progressive ataxia and cerebellar atrophy in brain MRI is suggestive of neuronal ceroid lipofuscinosis. If there is any specific clinical, or neuroimaging feature to suggest a particular inherited metabolic disease, appropriate metabolic investigations can be performed to support the diagnosis. The diagnosis is confirmed by targeted direct Sanger sequencing. If there is no specific clinical, biochemical, or neuroimaging feature, non-targeted genetic investigations including targeted next generation sequencing panel or whole exome or mitochondrial genome sequencing are applied to reach a diagnosis.

## Figures and Tables

**Table 1:** Inherited metabolic disorders presenting with ataxia are summarized by disease category, genetic defect, and clinical features.

Category	Disease Name	Gene	Clinical Features (Untreated or No Treatments)
Disorders of amino acid metabolism and transport	Maple syrup urine disease	*BCKDHA* *BCKDHB* *DBT*	GDD, ataxia (episodic or chronic), seizures, FTT, maple syrup odor
	Nonketotic hyperglycinemia	*GLDC* *AMT*	GDD, ataxia, seizures, hypotonia, spasticity
	HHH syndrome	*SLC25A15*	GDD, cognitive dysfunction, ataxia, spasticity, chronic liver dysfunction, mild or acute encephalopathy
	Sulfite oxidase deficiency	*SUOX*	GDD, movement disorder (episodic or chronic ataxia, dystonia, choreoathetosis), seizures, microcephaly, ectopia lentis
	L-2-hydroxyglutaric aciduria	*L2HGDH*	GDD, speech delay, ataxia, tremor, nystagmus, strabismus, seizures, macrocephaly
	Methylmalonic acidemia	*MCEE* *MMADHC*	GDD, movement disorder (ataxia, dysarthria), seizures, hypotonia, FTT, intermittent metabolic decompensation, vomiting, lethargy, hepatomegaly, hypothermia
	Glutaminase deficiency	*GLS*	GDD, movement disorder (ataxia, dysarthria), hypertonia
	Hartnup disease	*SLC6A19*	GDD, movement disorder (ataxia, dystonia, tremor), psychiatric abnormalities, skin rashes, nystagmus
**Disorders of carbohydrate metabolism**	Galactosemia	*GALT*	GDD, speech delay, ataxia, liver failure, bleeding, cataracts, premature ovarian failure
	Glucose transporter 1 deficiency	*SLC2A1*	GDD, speech delay, movement disorder (chronic or intermittent ataxia, dysarthria, dystonia, chorea, tremor), nystagmus, seizures, acquired microcephaly
**Disorders of mitochondrial energy metabolism**	Pyruvate dehydrogenase complex deficiency	*PDHA1* *PDHB* *DLAT* *PDP1*	GDD, intermittent ataxia, nystagmus, seizures, hypotonia, spasticity, microcephaly, peripheral neuropathy, encephalopathy
	Cerebral creatine deficiency syndromes GAMT deficiency CRTR deficiency	*GAMT* *SLC6A8*	GDD, cognitive dysfunction, speech delay, movement disorder (chronic or episodic ataxia, dystonia, chorea), seizures, behavioural disorder, hypotonia, dysmorphic features (*SLC6A8*)
	Dihydrolipoamide dehydrogenase deficiency	*DLD*	GDD, ataxia, tremor, seizures, hepatomegaly, liver dysfunction, vision impairment, microcephaly, hypotonia, spasticity
	Pyruvate carboxylase deficiency	*PC*	GDD, ataxia, seizures, hypotonia, FTT, metabolic acidosis, nystagmus
	Primary coenzyme Q10 deficiency	*COQ2* *COQ4* *COQ5* *COQ6* *COQ8A* *PDSS2* *ANO10*	GDD, movement disorder (ataxia, dystonia, parkinsonism), seizures, spasticity, hypotonia, myopathy, encephalopathy, stroke-like episodes, nephrotic syndrome, hypertrophic cardiomyopathy, retinopathy
	POLG related disorders	*POLG*	GDD, movement disorder (ataxia, chorea, parkinsonism), seizures, hypotonia, myopathy, psychiatric illness, stroke-like episodes, peripheral neuropathy, retinopathy, cataracts, hearing loss, liver involvement, endocrine dysfunction, cardiac involvement
	Leukoencephalopathy with brain stem and spinal cord involvement and lactate elevation	*DARS2*	GDD, cognitive dysfunction, motor decline, movement disorder (ataxia, dysarthria), seizures, spasticity
	TANGO2 related metabolic encephalopathy and arrhythmias	*TANGO2*	GDD, cognitive dysfunction, movement disorders (episodic ataxia, dysarthria), seizures, recurrent acute metabolic crises, rhabdomyolysis
	Optic atrophy type 1	*OPA1*	GDD, ataxia, proximal myopathy, visual impairment, vision loss, sensorineural hearing loss
	Optic atrophy type 10	*RTN4IP1*	GDD, cognitive dysfunction, ataxia, seizures, low vision
	FBXL4 disease	*FBXL4*	GDD, ataxia, seizures, lactic acidosis, FTT, hypotonia
	MELAS	*MT-TL1* *MT-ND5*	GDD, ataxia, seizures, stroke-like episodes, recurrent headaches, dementia, lactic acidemia, hearing impairment, peripheral neuropathy, ragged red fibers on muscle biopsy
	MERRF	*MT-TK* *MT-TF* *MT-TL1* *MT-TI* *MT-TP*	GDD, movement disorder (ataxia, myoclonus), ragged red fibers on muscle biopsy, lactic acidosis, hearing loss, neuropathy, dementia
	Leber hereditary optic neuropathy	*MT-ND4* *MT-ND6* *MT-ND1*	GDD, movement disorder (ataxia, postural tremor), myopathy, vision loss, optic atrophy, peripheral neuropathy
	NARP syndrome	*MT-ATP6* *MT-ND6*	GDD, cognitive dysfunction, ataxia, seizures, muscle weakness, retinopathy, dementia, neuropathy, hearing loss, cardiac conduction defects
	Infantile cerebellar-retinal degeneration	*ACO2*	GDD, movement disorder (ataxia, athetosis), seizures, FTT, hypotonia, optic atrophy, nystagmus, retinal dystrophy, microcephaly
	MNGIE syndrome	*TYMP*	GDD, ataxia, gastrointestinal dysmobility, cachexia, leukoencephalopathy, sensorimotor neuropathy, ptosis
	Kearns–Sayre syndrome	*mtDNA deletion*	GDD, cognitive dysfunction, ataxia, pigmentary retinopathy, cardiac conduction abnormality, progressive external ophthalmoplegia, hearing loss
	MTO1 disease	*MTO1*	GDD, ataxia, seizures, FTT, lactic acidosis, hypotonia
**Vitamin and cofactor responsive disorders**	Biotinidase deficiency	*BTD*	GDD, ataxia, seizures, hypotonia, skin rash, alopecia, conjunctivitis, hearing loss, vision problems
	Ataxia with vitamin E deficiency	*TTPA*	Progressive ataxia, dysdiadochokinesia, dysarthria, macular atrophy, retinitis pigmentosa, nystagmus
	Multiple carboxylase deficiency	*HLCS*	GDD, ataxia, seizures, hypotonia, FTT, vomiting, lethargy, metabolic ketolactic acidosis, skin rash
	Methylenetetrahydrofolate reductase deficiency	*MTHFR*	GDD, cognitive dysfunction, ataxia, seizures, psychiatric symptoms, hypotonia, spasticity, FTT, encephalopathy, microcephaly, apnea, myelopathy
	Riboflavin transporter deficiency neuronopathy	*SLC52A2 SLC52A3*	GDD, movement disorder (ataxia, tongue fasciculations), nystagmus, muscle weakness, FTT, respiratory insufficiency, nystagmus, sensorineural deafness, optic atrophy
	Cobalamin C deficiency	*MMACHA*	GDD, ataxia, tremor, nystagmus, seizures, hypotonia, FTT, nystagmus, pigmentary retinopathy
	Thiamine transporter deficiency	*SLC19A2* *SLC19A3*	GDD, movement disorders (recurrent ataxia, dystonia, dysarthria), nystagmus, external ophthalmoplegia, seizures, spasticity, eye movement abnormalities, encephalopathy, dysphagia, facial palsy
**Neurotransmitter disorders**	Succinic semialdehyde dehydrogenase deficiency	*ALDH5A1*	GDD, ataxia, seizures, strabismus, behavioural problems, hypotonia
**Organelle related disorders: lysosomal storage disorders**	Neuronal ceroid lipofuscinosis	*CLN1* *CLN2* *CLN5* *CLN6* *DNAJC5 MFSD8*	GDD, ataxia, seizures, spasticity, blindness, dementia, early death
	Alpha-mannosidosis	*MAN2B1*	GDD, cognitive dysfunction, ataxia, tremor, nystagmus, hypotonia, myopathy, psychiatric symptoms, distinct facial features, skeletal abnormalities, hearing loss, frequent infections
	Pompe disease	*GAA*	GDD, ataxia, hypotonia, hepatomegaly, respiratory insufficiency, cardiomegaly
	Fabry disease	*GLA*	GDD, ataxia, acroparesthesia, angiokeratoma, sweating abnormalities, corneal or lenticular opacity, cardiac disease, renal and cerebrovascular involvement
	Metachromatic leukodystrophy	*ARSA*	GDD, cognitive dysfunction, movement disorders (ataxia, dysarthria), seizures, psychiatric disturbance, hypotonia, spasticity, peripheral neuropathy, gallbladder involvement
	Fatty acid hydroxylase-associated neurodegeneration	*FA2H*	GDD, cognitive dysfunction, movement disorder (ataxia, dystonia, dysarthria), seizures, spasticity, optic atrophy or oculomotor abnormalities
	Gaucher disease type 2 Gaucher disease type 3	*GBA*	GDD, ataxia, hepatomegaly, splenomegaly, cytopenia, pulmonary involvement, stridor, oculomotor involvement, dysphagia
	Multiple sulfatase deficiency	*SUMF1*	GDD, ataxia, seizures, spasticity, vertebral abnormalities, skeletal deformities, dental abnormalities, cardiac manifestations, ophthalmic features
	Mucopolysaccharidosis type I (Hurler syndrome)	*IDUA*	GDD, ataxia, coarsened facial features, hepatosplenomegaly, progressive skeletal dysplasia, corneal clouding, hearing loss, cardiac involvement
	Sialuria	*GNE*	GDD, ataxia, neonatal jaundice, hepatomegaly, flat and coarse facial features, microcytic anemia, frequent upper respiratory infections
	Tay–Sachs disease	*HEXA*	GDD, movement disorders (ataxia, dystonia, tremor), seizures, spasticity, increased startle response, vision loss
	Sandhoff disease	*HEXB*	GDD, cognitive dysfunction, ataxia, seizures, spasticity, exaggerated startle response, cherry macules on eyes, splenomegaly, vision loss
	GM1 gangliosidosis	*GLB1*	GDD, movement disorder (ataxia, dystonia, parkinsonism, tremor), seizures, hypotonia, spasticity, cardiomyopathy, coarsened facial features, skeletal dysplasia
	Krabbe disease	*GALC*	GDD, ataxia, tremor, nystagmus, seizures, behavioural difficulties, hypotonia, spasticity, peripheral neuropathy, vision loss
	Sialidosis type I	*NEU1*	GDD, ataxia, seizures, cherry red macules, myoclonus, vision loss, corneal opacities
	Niemann–Pick type C disease	*NPC1* *NPC2*	GDD, movement disorder (ataxia, dystonia, dysarthria, tremor, gelastic cataplexy), vertical supranuclear gaze palsy seizures, psychiatric conditions, hypotonia, neonatal jaundice, hepatosplenomegaly, vertical supranuclear gaze palsy, dysphagia
	Salla disease	*SLC17A5*	GDD, cognitive dysfunction, movement disorder (ataxia, athetosis), seizures, hypotonia, spasticity, facial coarsening
**Organelle related disorders: peroxisomal disorders**	Zellweger spectrum disorders	*PEX2* *PEX10* *PEX12* *PEX16*	GDD, cognitive dysfunction, ataxia, nystagmus, seizures, hypotonia, sensorineural hearing loss, liver dysfunctions, bone stippling, retinal dystrophy
	X-linked adrenoleukodystrophy	*ABCD1*	GDD, ataxia, seizures, behaviour problems, vision loss, hearing loss
	Adult refsum disease	*PHYH* *PEX7C*	GDD, ataxia, anosmia, retinitis pigmentosa, peripheral neuropathy, hearing loss, ichthyosis, cardiac arrhythmias, skeletal abnormalities
**Organelle related disorders: golgi and pre golgi system disorders**	PMM2-CDG	*PMM2*	GDD, ataxia, nystagmus, strabismus, seizures, hypotonia, peripheral neuropathy, eye, skin, skeletal abnormalities, endocrine dysfunction
**Disorders of metal transport and metabolism**	Aceruloplasminemia	*CP*	GDD, cognitive dysfunction, movement disorder (ataxia, involuntary movement, dystonia, chorea, dysarthria, parkinsonism), retinal degeneration, diabetes mellitus, anemia
	Menkes disease	*ATP7A*	GDD, ataxia, seizures, hypotonia, kinky hair
	PKAN	*PANK2*	GDD, intellectual impairment, movement disorder (ataxia, dystonia, dysarthria, rigidity, choreoathetosis), spasticity, pigmentary retinal degeneration
	PLA2G6 disease	*PLA2G6*	GDD, cognitive dysfunction, movement disorder (ataxia in childhood phenotype, dystonia, parkinsonism), psychiatric symptoms (adult phenotype)
**Disorders of lipid and bile acid metabolism**	Mevalonate kinase deficiency	*MVK*	GDD, ataxia, nystagmus, FTT, lymphadenopathy, vision problems, hepatosplenomegaly, abdominal pain
	Abetalipoproteinemia	*MTTP*	Ataxia, dysarthria, FTT, progressive vision loss, muscle weakness
	Cerebrotendinous xanthomatosis	*CYP27A1*	GDD, movement disorders (ataxia, dystonia, parkinsonism), seizures, psychiatric disturbances, diarrhea, cataracts, xanthomas, dementia, peripheral neuropathy
**Disorders of nucleic acid and heme metabolism**	Phosphoribosylpyrophosphate synthetase superactivity	*PRPS1*	GDD, cognitive dysfunction, ataxia, hypotonia, hyperuricemia, hyperuricosuria, urinary stone, gouty arthritis, sensorineural hearing loss
	Purine nucleoside phosphorylase deficiency	*PNP*	GDD, cognitive dysfunction, ataxia, spasticity, increased risk of autoimmune disorders, recurrent infections

Abbreviations:.CRTR = creatine transporter; FBXL4 = F-Box and Leucine- Rich Repeat Protein 4; FTT = Failure to thrive; GAMT= guanidinoacetate methyltransferase; GDD = global developmental delay; HHH = hyperornithinemia hyperammonemia homocitrullinuria; MELAS = mitochondrial encephalomyopathy, lactic acidosis, and stroke-like episodes; MERRF = myoclonic epilepsy with ragged-red fibers; MNGIE = mitochondrial neurogastrointestinal encephalopathy; MTO1 = mitochondrial tRNA translation optimization 1; NARP = neuropathy, ataxia, and retinitis pigmentosa; PKAN = pantothenate kinase-associated neurodegeneration; PLA2G6 = Phospholipase A2 Group VI; PMM2-CDG = Phosphomannomutase 2-Congenital disorder of glycosylation; POLG = Plymerase Gamma; TANGO2 = Transport and golgi organization.

**Table 2:** Metabolic investigations for inherited metabolic disorders causing ataxia.

Investigations	Type of Investigations	Inherited Metabolic Disorders
Blood metabolic investigations	Ammonium	MMA
	Lactate	Mitochondrial disorders
	Plasma amino acids	MSUD, NKH, HHH syndrome, glutaminase deficiency
	Biotinidase activity	Biotinidase deficiency
	Homocysteine	CblC deficiency, MTHFR deficiency
	Acylcarnitine profile	MMA
	Glucose (paired with CSF glucose)	GLUT1 deficiency
	Pyruvate	Pyruvate dehydrogenase complex deficiency, mitochondrial disorders
	Enzyme assays for lysosomal storage disorders in WBC	Disease specific enzyme activity measurements
	VLCFA	Zellweger spectrum disorders, X-ALD
	Transferrine isoelectric focusing	*PMM2-*CDG
	Copper	Menkes disease
	Ceruloplasmin	Aceruloplasminemia, Menkes disease
	Phytanic acid	Refsum disease
	Vitamin E	Ataxia with vitamin E deficiency
	LDL-cholesterol, triglyceride, apolipoprotein (apo) B	Abetalipoproteinemia
	Galactose-1-phosphate uridylyl transferase activity	Galactosemia
Urine metabolic investigations	Amino acids	Hartnup disease
	Organic acids	MMA, SSADH deficiency, mevalonate kinase deficiency
	Sulfocysteine	SOD
	Oligosaccharides	Alpha-mannosidosis
	Glycosaminoglycan	MPS
	Free and total sialic acid	Salla disease
	Guanidinoacetate	GAMT deficiency AGAT deficiency
	Creatine to creatinine ratio	Creatine transporter deficiency
CSF	Glucose	GLUT1 deficiency
	Lactate	GLUT1 deficiency, PDH complex deficiency, mitochondrial disorders
	Amino acids	NKH
	Neurotransmitters	Inherited neurotransmitter disorders
	MTHF	Methylenetetrahydrofolate reductase deficiency
	GABA (total and free)	SSADH deficiency
Muscle biopsy	Muscle histology	Mitochondrial disorders
	Muscle electron microscopy	Mitochondrial disorders
	Respiratory chain enzyme activity meausurements	Mitochondrial disorders
	Coenzyme Q10 measurement	Co-enzyme Q10 deficiency
Skin fibroblasts	Respiratory chain enzyme activity measurement	Mitochondrial disorders
	Pyruvate dehydrogenase activity measurement	Pyruvate dehydrogenase complex deficiency
	Pyruvate carboxylase activity measurement	Pyruvate carboxylase deficiency
Molecular genetic investigations	Targeted next generation sequencing panel for	Leigh disease, mitochondrial disorders, ataxia
	Whole exome sequencing	Non-targeted molecular genetic investigation
	Mitochondrial genome sequencing	Non-targeted molecular genetic investigation

Abbreviations: AGAT = Arginine:glycine amidinotransferase; cblC def = Cobalamin C deficienc; CSF = Cerebrospinal fluid; GABA = gamma-Aminobutyric acid; GAMT = guanidinoacetate methyltransferase; GLUT1 def = Glucose transporter 1 deficiency; HHH = hyperornithinemia hyperammonemia homocitrullinuria; MMA = methylmalonic acidemia; MPS = Mucopolysaccharidosis; MSUD = Maple syrup urine disease; MTHFR def = Methylenetetrahydrofolate reductase deficiency; NKH = Non-ketotic hyperglycinemia; PDH = Pyruvate dehydrogenase; PMM2-CDG= Phosphomannomutase 2-Congenital disorder of glycosylation; SOD = Sphincter of Oddi Dysfunction; SSADH Def = Succinic semialdehyde dehydrogenase deficiency; VLCFA = very long chain fatty acids; WBC = White Blood Cell; X-ALD = X-linked adrenoleukodystrophy.

**Table 3:** Inherited metabolic disorders with specific treatments to amenable disease outcomes and their biochemical and neuroimaging features are summarized in Table 2.

Disease Name	Biochemical Features	Neuroimaging	Treatments
Maple syrup urine disease	↑ leucine, alloisoleucine, isoleucine, valine in plasma amino acid analysis ↑ ketones and metabolic acidosis during acute metabolic decompensation	Diffusion restriction in cerebellum, WM, BS, BG	Leucine-restricted diet, medical formula, thiamine Branched chain amino acid diet restriction
Hartnup disease	↑ neutral amino acids (alanine, serine, threonine, valine, leucine, isoleucine, phenylalanine, tyrosine, tryptophan, histidine, citrulline, asparagine, glutamine) in urine amino acid analysis	Diffuse brain atrophy	Nicotinamide, neomycin, tryptophan ethyl ester, tryptophan rich protein intake
Riboflavin transporter deficiency neuronopathy	Abnormal acylcarnitine profile (elevated short, medium or long chain species)	Normal to cerebellar atrophy, increased T2 intensity in brain stem, cerebellum	Riboflavin
Biotinidase deficiency	↓ Serum biotinidase activity ↑ 3-methylcrotonylglycine, 3-hydroxyisovaleric acid, methylcitrate, 3-hydroxypropionate in urine organic acid analysis Metabolic ketolactic acidosis Hyperammonemia	Cerebral or cerebellar atrophy, delayed myelination	Biotin
Multiple carboxylase deficiency	↑ hydroxypentanoylcarnitine ↑ 3-methylcrotonylglycine, 3-hydroxyisovaleric acid, methylcitrate, 3-hydroxypropionate in urine organic acid analysis Metabolic ketolactic acidosis	Cerebral atrophy, delayed myelination	Biotin
Thiamine transporter deficiency	Sometimes ↑ CSF and blood lactate	Atrophy of caudate and putamen, swelling of pons	Biotin, thiamine
Methylenetetrahydrofolate reductase deficiency	↑ plasma homocysteine ↓ to normal methionine in plasma amino acid analysis	Brain atrophy, increased WM signal in T2	Betaine, folic acid, methionine, pyridoxine, carnitine, 5-methyltetrahydrofolate
Cobalamin C deficiency	↑ plasma homocysteine ↓ to normal methionine in plasma amino acid analysis ↑ methylmalonic acid in urine organic acid analysis	Brain atrophy, WM edema	Hydroxocobalamin, betaine, carnitine, folic acid
Galactosemia	↑ erythrocyte galactose-1-phosphate ↓ erythrocyte GALT activity	Cerebellar and cerebral atrophy, delayed myelination	Galactose and lactose free diet, vitamin D, calcium
Glucose transporter 1 deficiency	↓ CSF glucose with normal blood glucose ↓ erythrocyte 3-O-methyl-D-glucose uptake	Normal	Ketogenic diet
Cerebral creatine deficiency syndromes	↑ urine, plasma GAA (*GAMT* deficiency) ↑ urine creatine to creatinine ratio	Normal to increased T2 signal in BG	*GAMT* deficiency: creatinine, ornithine, arginine restricted diet *CRTR* deficiency: arginine, glycine, creatine
Primary coenzyme Q10 deficiency	↓ coenzyme Q10 in skeletal muscle ↓ complex I+III and II+III activity in muscle	Cerebellar atrophy, and increased T2 signal intensity cerebellum	Coenzyme Q10
Cerebrotendinous xanthomatosis	↑ cholestanol in plasma ↓ to normal plasma cholesterol	Diffuse brain atrophy, increased signal intensity in WM, substantia nigra, spinal cord in T2	Chenodeoxycholic acid
Niemann-pick type C disease	↑ oxysterols in plasma Positive filipin staining in cultured fibroblasts	Cerebral and cerebellar atrophy, increased WM intensity in T2	Miglustat
Pyruvate dehydrogenase complex deficiency	↑ Blood and CSF lactate ↑ Blood and CSF pyruvate and alanine Normal lactate to pyruvate ratio	Cerebral and cerebellar atrophy, increased signal in striatum and thalamus in T2	Thiamine, carnitine, lipoic acid, ketogenic diet
Dihydrolipoamide dehydrogenase deficiency	↑ Blood and CSF lactate ↑ Blood and CSF pyruvate and alanine ↑ alpha ketoglutarate in urine organic acid analysis ↑ leucine, valine, isoleucine, alloisoleucine in plasma amino acid analysis	Increased signal intensity in BG in T2	Thiamine, ketogenic diet
HHH syndrome	↑ ammonia ↑ ornithine in plasma amino acid analysis ↑ homocitrulline in urine amino acid analysis	Cerebral atrophy, increased WM signal, increased BG signal, stroke-like lesions	Citrulline, arginine, sodium phenylbutyrate, protein restricted diet
Adult refsum disease	↑ plasma phytanic acid	Normal or cerebral atrophy	Phytanic acid restricted diet
Aceruloplasminemia	↓ serum ceruloplasmin ↓ serum copper or iron ↑ serum ferritin ↑ hepatic iron	Decreased signal intensity in BG in T2	Iron chelating agents (desferrioxamine, deferiprone, or deferasirox), combined IV desferrioxamine and fresh-frozen human plasma (FFP)
Pyruvate carboxylase deficiency	↑ lactate Normal lactate to pyruvate ratio ↑ alanine, citrulline, lysine in plasma and urine amino acid analysis ↓ aspartic acid, glutamine in plasma and urine amino acid analysis	Hypomyelination, cysts in cortex, BG, brain stem and, cerebellum	Acute management: IV glucose Chronic management: citrate, aspartate, biotin, liver transplantation
Alpha-mannosidosis	↓ alpha-mannosidase activity	Cerebral and cerebellar atrophy	Velmanase alfa (where approved)
Fabry disease	↓ alpha-galactosidase A activity ↑ globotriaosylsphingosine in urine and plasma	Cerebral atrophy, increased signal intensity in WM in T2, stroke-like lesions	Agalsidase beta
Neuronal ceroid lipofuscinosis type 2 *CLN2* disease	↓ tripeptidyl peptidase 1 activity	Cerebral and cerebellar atrophy, dark thalami in T2	Cerliponase alfa intracerebroventricular
Mucopolysaccharidosis type I (Hurler syndrome)	↓ alpha-L-iduronidase activity ↑ urinary glycosaminoglycans ↑ heparan dermatan sulfate in urine glucose amino glucan analysis.	Cerebellar hypoplasia	HSCT Laronidase (for non-CNS manifestations)
Krabbe disease	↓ galactocerebrosidase activity	Cerebral atrophy, demyelination in brain stem and cerebellum, chiasmatic enlargement	HSCT
Metachromatic leukodystrophy	↓arylsulfatatase A activity ↑sulfatides in urine	Cerebral atrophy, demyelination in brain stem and cerebellum, chiasmatic enlargement	HSCT
X-linked adrenoleukodystrophy	↑ VLCFA in plasma	Symmetric enhanced T2 signal in the parieto-occipital region with contrast enhancement at the advancing margin	HSCT
Ataxia with vitamin E deficiency	↓ vitamin E level	Cerebellar atrophy, small T2 high-intensity spots in the periventricular region and the deep white matter	Oral vitamin E supplementation
Abetalipoproteinemia	↓LDL-cholesterol, triglyceride, and apolipoprotein (apo) B	Delayed myelination	Low-fat diet, essential fatty acid supplementation, fat soluable vitamin supplementation (ADEK)

Abbreviations: BG = basal ganglia; CLN2 = Neuronal ceroid lipofuscinosis type 2; CRTR = Creatine Transporter; CSF = cerebrospinal fluid; GAA= guanidinoacetate; GALT = galactose-1-phosphate uridylyltransferase; GAMT = guanidinoacetate methyltransferase; HHH = hyperornithinemia hyperammonemia homocitrullinuria; HSCT = hematopoietic stem cell transplant; VLCFA = very long chain fatty acids; WM = white matter. ↑: elevated; ↓: Decreased (low).

## Abbreviations

ABCD1	ATP-binding cassette domain 1
AGAT	Arginine:glycine amidinotransferase
BCKD	Branched-chain alpha-ketoacid dehydrogenase complex
BVVL	Brown-Vialetto-Van Laere
CDG	Congenital disorders of glycosylation
CRTR	Creatine transporter
CSF	Cerebrospinal fluid
FAD	Flavin adenine dinucleotide
FL	Fazio-Londe
FMN	Flavin mononucleotide
GALT	Galatose-1-phosphate uridylyltransferase
GAMT	Guanidinoacetate N-methyltransferase
GLUT1	Glucose transporter 1
HSCT	Hematopoietic stem cell transplantation
NCL	Nneuronal ceroid lipofuscinoses
PDHC	Pyruvate dehydrogenase complex deficiency
MSUD	Maple syrup urine disease
X-ALD	X-linked adrenoleukodystrophy
